# Assessing Lexical and Syntactic Comprehension in Deaf Signing Adults

**DOI:** 10.1093/deafed/enad022

**Published:** 2023-07-31

**Authors:** Giorgia Zorzi, Valentina Aristodemo, Beatrice Giustolisi, Charlotte Hauser, Caterina Donati, Carlo Cecchetto

**Affiliations:** Department of Translation and Language Sciences, Universitat Pompeu Fabra, Barcelona, Spain; Department of Language, Literature, Mathematics and Interpreting, Western Norway University of Applied Sciences, Bergen, Norway; Dipartimento di Psicologia e Scienze Cognitive, Università di Trento, Italy; Université Toulouse - Jean Jaurès, UMR 5263 CLLE, France; Department of Psychology, University of Milano-Bicocca, Milan, Italy; Université Paris 8, CNRS, Structures Formelles du Langage UMR 7023, Paris, France; Université Paris Cité, CNRS, Laboratoire Linguistique Formelle UMR 7110, Paris, France; Department of Psychology, University of Milano-Bicocca, Milan, Italy; Université Paris 8, CNRS, Structures Formelles du Langage UMR 7023, Paris, France

## Abstract

Among the existing sign language assessment tools, only a small number can be used in clinical settings. This contribution aims at presenting three comprehension assessment tests (two lexical and one syntactic) that offer a solid basis to build tools to assess language impairments in deaf signing adults. We provide the material and guidelines, based on psychometric analyses of the items, to make these tests suitable for clinical assessment. They are available for French Sign Language and Italian Sign Language. So far, the three tests were administered to three groups of deaf participants based on age of exposure (AoE) to sign language: native (AoE from birth), early (AoE = from 1 to 5 years), and late (AoE = from 6 to 15 years) signers. The results showed that the three tests are easy for the typical deaf signing population, and therefore, they can be adapted into tests that assess a deaf signing population with language impairments. Moreover, the results of the syntactic test reveal a categorial difference between native and non-native signers and therefore show the need for baselines that mirror the effect of AoE to sign language when assessing language competence, in particular in clinical assessment.

## Introduction

The vast majority of existing sign language assessment tools are meant to study language acquisition in deaf signing children, for educational purposes or for linguistic research, and very few can also be used to assess deaf signing adults (Haug, 2005). More importantly, only a few of them eventually plan intervention for developmental language impairments and there is still an important need for sign language assessment tests that can detect language disorders (Hauser et al., 2015). There is also a general lack of tools that incorporate clinical neuropsychological practices detecting deficits that might interfere with typical language processing (i.e., deficits in attention, learning, or emotional difficulties), which could presumably result in poor language development (Hauser et al., 2015; Quinto-Pozos et al., 2014).

The goal of this paper is two-folded: (i) present three comprehension tests (two lexical and one syntactic) that can be adapted to assess deaf signing adults with language impairments and (ii) path the way to turn them into clinical tests by selecting the material, on the basis of psychometric analyses of the items. The three tools that will be presented were built in the framework of the H2020 SIGN-HUB project to specifically test native and non-native deaf signers. These language-specific instruments have been built for different sign languages, but in this work, we will focus on French Sign Language (LSF) and Italian Sign Language (LIS).

In this paper, we will first provide a general overview of language assessment tools in sign languages, focusing in particular on those that can be used in clinical settings. We shall then present the three SIGN-HUB tests that have the potential to become reliable clinical tools for deaf impaired signers of LSF and LIS. We will describe their characteristics and the results of their administration to a population with different ages of exposure (AoEs) to sign language of about 45 healthy deaf adult signers for each sign language. Based on these results, we will point out the aspects that indicate that these tests could easily be adapted to be used in clinical settings. We will also provide the results of psychometric analyses of the items and give indications on how to modify the tests to make them suitable to be applied to an impaired population. All the materials to build the tests are also made available through the OSF page of the present project. Lastly, based on the suggestions of the present results and that of a number of previous studies targeting the same sign languages (e.g., Aristodemo et al., 2022; Hauser et al., 2021), we will conclude by underlining the need to establish different baselines based on the AoE when assessing the syntax of a deaf signing population, including in clinical settings. The results of the syntactic test presented in this study support the claim that there is a categorical difference between signers exposed from birth to a sign language and signers exposed to it at any point later in life. We will then stress that, when looking at language competence in clinical settings, it is crucial to establish baselines mirroring the grammar of native and non-native signers since a good understanding of the individual's likely baseline would affect the determination of whether an acquired language impairment is present.

### Sign Language Assessment Tools: A Focus on Clinical Tests

The sign language assessment tools that have been developed so far focus on three main areas: language acquisition, education, and linguistic research (Haug, 2005). The tests of the first type aim at assessing language skills in hearing and deaf children, either first and second language learners or bilingual, identifying language difficulties and evaluating possible interventions to improve the level of language learning. Some examples are the *American Sign Language-Proficiency Assessment* (ASL-PA; Maller et al., 1999), the *Assessing British Sign Language Development Receptive Skills test* (BSL-RST; Herman et al., 1999), and the *Aachen Test for Basic German Sign Language Competence* (ATG; Fehrmann et al., 1995a, 1995b).

The tools meant for educational purposes investigate the level of specific linguistic structures in deaf children and can be used to make a diagnostic, in addition to helping educators develop strategies for teaching. The two main tests in this category are the *American Sign Language Assessment Instrument* (ASLAI; Hoffmeister, 1994, 1999, 2000) and the *Test of ASL* (TASL; Strong & Prinz, 1997, 2000).

In the third group, concerning linguistic research, we find tests that have the goal of gathering data on specific morphosyntactic structures, in particular studying the effects of age of acquisition in the grammatical processing of a sign language. Among others, there are the *Test Battery for ASL Morphology and Syntax* (Supalla et al., 1995) and the *Grammatical Judgment Test for ASL* (Boudreault, 1999; Boudreault & Mayberry, 2000). In the three areas, both comprehension and production are tested. Most of the tests developed for the first two types assess only children (overall age range: 2–16 y.o.), with the exception of the *Aachen Test for Basic German Sign Language Competence*, which also targets adults: hearing parents of deaf children and hearing professionals working with German Sign Language such as interpreters and teachers, among others. Native and non-native deaf signer teenagers and adults are mainly targeted in the linguistic tests (overall age range: 3–84 y.o.), depending on the purpose of the study.

An important test that targets adults, though, is the *American Sign Language Comprehension Test* (ASL-CT; Hauser et al., 2016). The ASL-CT has the innovative feature of immediately providing a score on ASL proficiency without relying on raters. It is a multiple-choice test that measures ASL receptive skills and assesses grammatical aspects of ASL including phonology, vocabulary, role shift, and depicting constructions (Dudis, 2004; Liddell, 2003).

#### Issues in adapting assessment tests

Haug and Mann (2008) underline that there are several aspects to consider when selecting an assessment test, other than the age range they target. It is in fact crucial to look, among other features, at whether the test has a background based on linguistic research, if it is language specific or if it was adapted from an existing test, if it is valid, and if it is reliable. They also point out that it is common practice to adapt a test from a sign language to another and this often leads to issues related to changes of items and design. The adaptation of established psychometric properties of the source test to a new version might also put the new test at risk of failing its purpose. When adapting a test to a new target language, it is crucial to develop a test that is as close as possible to its source. The goal is to retain the measurements of the original test but making the changes that are necessary to respect the linguistic and cultural constraints of the target language (Oakland & Lane, 2004). A general lack of linguistic research on the structures investigated in the tests makes this process even more difficult, especially when language-related differences might require a change in the design of the test (Haug & Mann, 2008).^1^ This affects the psychometric properties of such tests, that is, their validity and reliability.

The validity of a test consists in whether it actually measures the construct it intends to measure, and its reliability is “the degree of stability of measurement that exists when a measurement is made repeatedly under different conditions or by different observers” (Law et al., 1998). The reliability of a test can be measured over time through a test–retest (Kline, 2000), for which scores of the same participants obtained on two different occasions are correlated. Another important aspect of a test is its standardization, which depends on ​​the size of the population represented by the sample and the homogeneity or heterogeneity of the population (Kline, 2000). Content validity, which relates to the adequacy of the sampling of the content to be measured, should be achieved through the collaboration with deaf sign language experts if they are not present in the research team.

#### Age of exposure and availability of assessment tests for adults

The age when each participant has first been in contact with the target sign language is an important variable, which is, however, targeted only by linguistic tests.

It has been documented since the ‘90s that early exposure to sign language is crucial to language acquisition (Mayberry et al., 2002), with studies showing that non-native signers differ from native signers in several morpho-syntactic tasks (e.g., Emmorey & Corina, 1990; Emmorey et al., 1995; Mayberry & Eichen, 1991; Mayberry, 1993; Boudreault & Mayberry, 2006; Cormier et al., 2012a). These considerations are even more important when assessing deaf adults in clinical environments. The current situation on the assessment of deaf signing adults with language impairments is that the availability of specific tools is extremely scarce, making it difficult to plan clinical intervention. Very few tests are created with the goal of becoming clinical assessment tools.

The general picture regarding the existent assessment tools in sign language is problematic, even for well-studied sign languages such as American Sign Language (ASL) and British Sign Language (BSL), but even more so for LSF and LIS, which are the target languages of this paper. For ASL, for example, most of the existing tests are unavailable for distribution because they require specialized researchers (Hauser et al., 2015) and even fewer are commercially available for professionals (Secora et al., 2022). Moreover, the majority of the tests used even for clinical purposes for deaf adults focus mostly on production. A few examples are the *Detection Test for Language Impairments in Adults and the Aged* (Macoir et al., 2017), the *American Sign Language–Sentence Reproduction Test* (ASL-SRT; Hammill et al., 1994), and the *Depiction Comprehension Test* (DCT; Brookshire & Nicholas, 1993), among others.

An important exception is the battery of four assessment tests in BSL discussed in Atkinson et al. (2005): *The Sign to Picture Matching test*, *The BSL verbs and sentences test*, *The BSL locatives test*, and *The Classifiers: Placement, Orientation and rotation test.* All these tests were administered to left hemisphere and right hemisphere brain-damaged signers.

**Figure 1:**
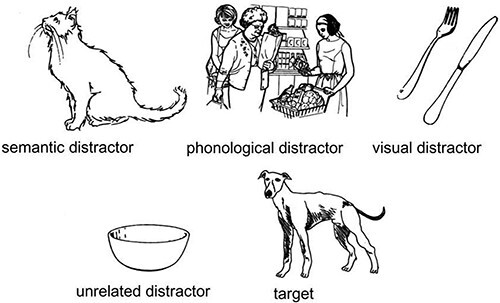
Example of the images used for an item in The Sign to Picture Matching test (adapted from Atkinson et al., 2005: 237). Reprinted from Brain and Language, 94(2), Atkinson, J., Marshall, J., Woll, B., & Thacker, A., Testing comprehension abilities in users of British sign language following CVA, 233–248., Copyright (2005), with permission from Elsevier.

**Figure 2:**
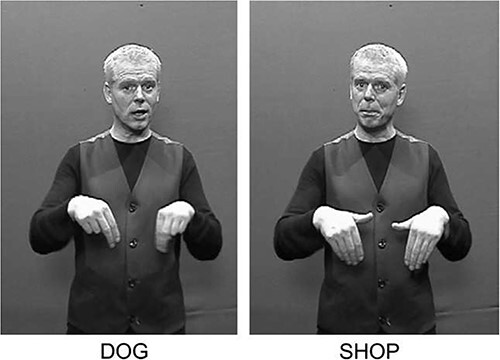
Example of the target dog in British Sign Language (BSL) and its phonological distractor shop from The Sign to Picture Matching test (Atkinson et al., 2005: 238). Reprinted from Brain and Language, 94(2), Atkinson, J., Marshall, J., Woll, B., & Thacker, A., Testing comprehension abilities in users of British sign language following CVA, 233–248., Copyright (2005), with permission from Elsevier.

*The Sign to Picture Matching test* assesses the comprehension of nouns. Participants watch the examiner producing a sign and have to choose the correct image among five pictures containing the target, a phonological distractor, a semantic distractor, a visual distractor, and an unrelated distractor. Figure 1 illustrates an example of an item of the test in which the target sign is dog. The phonological distractor is shop, which forms a minimal pair for handshape with the target sign (both signs dog and shop are shown in Figure 2); the semantic distractor is cat. The visual distractor, designed to detect people who might use iconic strategies in comprehension, is cutlery because the sign dog resembles the action of holding a knife and a fork. Lastly, the unrelated distractor is semantically related to the visual distractor. This test contains 40 items (20 iconic and 20 non-iconic). Iconicity was rated by 21 deaf signers on a scale from 1 to 7.

The *BSL verbs and sentences test* assesses the comprehension of verbs and sentences focusing on morphologically marked and unmarked verbs and reversible and non-reversible sentences. Participants watch a signed verb phrase or sentence produced by the examiner and have to choose the matching picture among four.

The *BSL locatives test* assesses comprehension of spatial relationships conveyed by classifiers and prepositional constructions. The examiner signs a locative sentence and participants have to choose the matching picture among four.

The *Classifiers: Placement, Orientation and rotation test* assesses the ability to process the object denoted by a classifier and the additional spatial information. The examiner signs a single classifier, which has to be matched to one of the four pictures.

All these tests were administered to a deaf control group including natives and early and late signers. However, the authors do not take into account AoE to sign language as a main factor in the analysis.^2^

Given the limits just described of sign language assessment tools to be used in clinical settings with deaf signing adults, one of the main goals of the SIGN-HUB project was to contribute to filling this gap by designing several assessment tools with the potential to be used with deaf adults with language impairments. Particular importance was given to the impact of AoE by assessing deaf native, early and late signers. Importantly, these tools had the ultimate goal to be freely available to clinical practitioners.

### SIGN-HUB Assessment Tests

The SIGN-HUB project tests were specifically designed for a number of European sign languages. For each language, a group of deaf consultants, all native signers, worked closely with the research team to select all materials, hence ensuring content validity (Haug, 2005). The Italian research team included a deaf researcher. The tests aimed at assessing lexical and morphosyntactic competence in different populations of deaf signers with three general goals: (i) investigate the impact of AoE in adult deaf signers’ linguistic competence, (ii) contribute to the comparative analysis of some specific linguistic phenomena, and (iii) select potential clinical assessment tools.

Focusing specifically on goal iii, for lexical assessment, we developed tests that would be sensitive to the phonological and semantic errors that participants can make. The rationale was that once we can identify the different types of errors in signers, we will be able to diagnose the type of impairment and recommend treatment that is targeted at the impaired component. A similar rationale held for the syntactic assessment: We developed assessment tools that target linguistic structures that are known to be affected in case of language impairments (i.e., relative clauses and *wh-*questions), as in Friedmann et al. (2009). In addition to this, we identified syntactic structures that are sign-language specific, notably those involving the grammatical use of space (including role shift and agreement verbs, also called directional verbs).^3^

Among the tests developed within the SIGN-HUB project, the *Lexical comprehension task with phonological distractors* (Zorzi et al., 2019b, 2019c for LSF and LIS, respectively), the *Lexical comprehension task with semantic distractors* (Zorzi et al., 2019a, 2019d for LSF and LIS, respectively) and the *Verbal agreement comprehension task* (Aristodemo et al., 2019 for LSF, Sala et al., 2019 for LIS) are the tests that have the potential to become clinical assessment tools for sign language assessment in adults with language impairments because of the very good performance of the healthy population. The two lexical tests are sign-to-picture matching tasks that assess comprehension against phonological distractors and semantic distractors, respectively. The syntactic test is a truth-value judgment task that assesses the comprehension of agreement verbs.

For each language, all three tests were administered to a population of about 45 healthy deaf adult signers with different ages of exposure to sign language.

The materials and data underlying this article are available in an OSF repository at https://osf.io/njzse/.

#### Participants

In the SIGN-HUB tests, participants were selected following three general criteria of inclusion: (i) onset of deafness not later than 3 years of age; (ii) first exposure to sign language not later than 15 years of age; and (iii) the target sign language as their preferred mean of communication. In order to investigate the impact of AoE in language comprehension, participants were divided into three groups: (i) native (exposed to sign language from birth (AoE = 0) and having at least one deaf signing parent), (ii) early signers (AoE = 1–5 years of age), and (iii) late signers (AoE = 6–15 years of age). Despite skepticism in the recent literature about the notion and importance of native speakers or signers (Cheng et al., 2021; Zorzi et al., 2022), participants were divided into these three groups because one of the main goals was to verify the effects of the AoE to language. All participants also took an *Odd One Out Cognitive Task* (cf. Aristodemo & Friedmann, 2019 for LSF; Giustolisi & Friedmann, 2019 for LIS). The task consisted in finding the intruder in each of 28 sets of four pictures, and it was designed to detect potential cases of severe cognitive impairment. For each participant, z-scores were calculated considering language group mean and standard deviations. Participants with z-scores lower than −2.5 were excluded from the study. One LIS native signer with a z-score of −3.94 was excluded from the LIS pool. No participant was excluded from the LSF pool. The final number of participants consisted therefore of 44 LIS signers and 43 LSF signers (Table 1).

**Table 1:** Participants’ characteristics per group and language adapted from Zorzi et al. (2022)

Group	SL	N.	Age	AoE	Everyday use of SL	Deaf parent(s)	Signing parent(s)	Context of exposure to SL	Years of SL experience
NATIVE	LIS	16	Range: 30–60 yrs M: 43 yrs	0	16	16	16	Family: 16	30–60 (M = 43)
	LSF	14	Range: 26–54 yrs M: 39 yrs	0	13^8^	13	13	Family: 13 (1 NS)	26–54 (M = 39)
EARLY	LIS	15	Range: 34–62 yrs M: 48 yrs	2–5 yrs (M: 3,9)	13	1	3	Family: 4 Preschool: 10 (1 NS)	32–58 (M = 47)
	LSF	15	Range: 24–47 yrs M: 34 yrs	1–5.5 yrs (M: 3.4)	10	none	1	Family: 3 Preschool: 11 (1 NS)	20–39 (M = 30)
LATE	LIS	13	Range: 40–65 yrs M: 50 yrs	6–15 yrs (M: 9.1)	11	none	1	Family: 2 School: 9 (2 NS)	26–58 (M = 41)
	LSF	14	Range: 19–72 yrs M: 40 yrs	6–14 yrs (M: 9.2)	11	2	1	Family: 1 School: 9 (4 NS)	9–63 (M = 31)

Additional information displayed in Table 1 was collected through a questionnaire that participants filled in before taking part in the tests.

Within the SIGN-HUB project, data collection spanned over a period of about 3 months, with two sessions per participant during which participants performed the three tests presented in the present paper and several other tests. In a few cases, some participants missed one experimental session; therefore, the total number of participants slightly differs across tasks.

In the three tasks reported in the present paper, the total number of participants was 42 for LIS and 43 for LSF. As for the *Verbal agreement comprehension task*, the final analysis was performed on data from only 39 LIS and 40 LSF signers because 3 LIS and 3 LSF signers were excluded due to a low score in the control items (below 75% accuracy).

#### Lexical comprehension tests

Sign comprehension involves both phonological and semantic processing. When seeing a sign, phonological representations close to that of the sign might get activated, and the same happens with the semantic concepts related to this sign.

To selectively detect potential phonological and semantic deficits, we built two different sign-to-picture matching tests for each language: one with phonological distractors and one with semantic distractors. Specifically, each target picture appeared together with pictures corresponding to phonological neighbors or to semantically related items, respectively. The idea behind the construction of two different tests was that in a clinical setting, if the subject has a phonological deficit, they are expected to display a significantly lower performance on the test with phonological distractors compared to the test with semantic distractors. On the contrary, in case of a semantic deficit, the expectation is the opposite: more errors in the task with semantic distractors compared to the task with phonological distractors.

In both tests, items were selected following three criteria: (a) minimize regional variation; (b) avoid “extreme transparency”; and (c) representability with a picture. Proper names, classifiers, and compounds were not included mainly due to (a) and (c).^4^

##### Lexical Comprehension With Phonological Distractors

The *Lexical comprehension task with phonological distractors* (cf., Zorzi, Aristodemo, Friedmann, & Cecchetto and C., Donati, C., 2019; Zorzi, Giustolisi, Cecchetto, & Donati, 2019 for LSF and LIS, respectively) is a sign-to-picture matching task. The target picture was displayed together with five more pictures: three corresponding to signs that form a minimal pair with the given sign and two corresponding to more loosely phonologically related distractors. For the collection of the minimal pairs for each target sign, the usual definition of minimal pairs was used, where two signs with a different meaning differ only by one parameter, considering handshape, location, orientation, and movement. Among the minimal pairs, we aimed at selecting for each item one minimal pair for handshape, location, and movement. Very often, though, this was not possible, and a target could end up with more than one minimal pair for the same parameter. As for the loosely phonologically related distractors, signs were differing from the target in more than one parameter. We also controlled that no distractor was in the same semantic category of the target. The LIS test contained 22 items and the LSF contained 25. Items selection was difficult due to the selection criteria mentioned above. Moreover, for LIS, minimizing regional variation was especially challenging. If a sign selected as a target had more than three recognized sign variations, this item was not retained for the test. Such a problem did not occur for the selection of LSF items, where lexical variation is more reduced.

**Figure 3:**
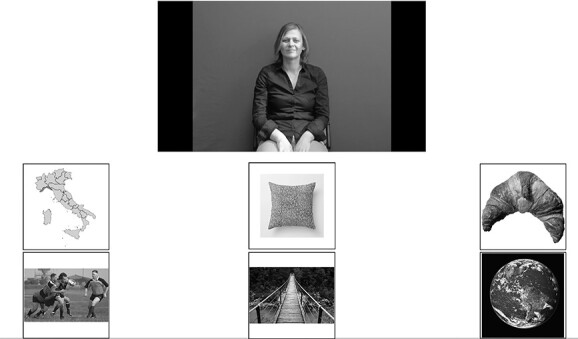
Example of one item of the lexical comprehension task with phonological distractors in Italian Sign Language (LIS) (the original pictures in the test were in color).

Each participant watched a video displaying the target sign while the six pictures were displayed. The participant had to click on the matching picture (see Figure 3). The video could be watched only once. The test started with one training item. No filler was introduced.

##### Lexical Comprehension With Semantic Distractors

**Figure 4:**
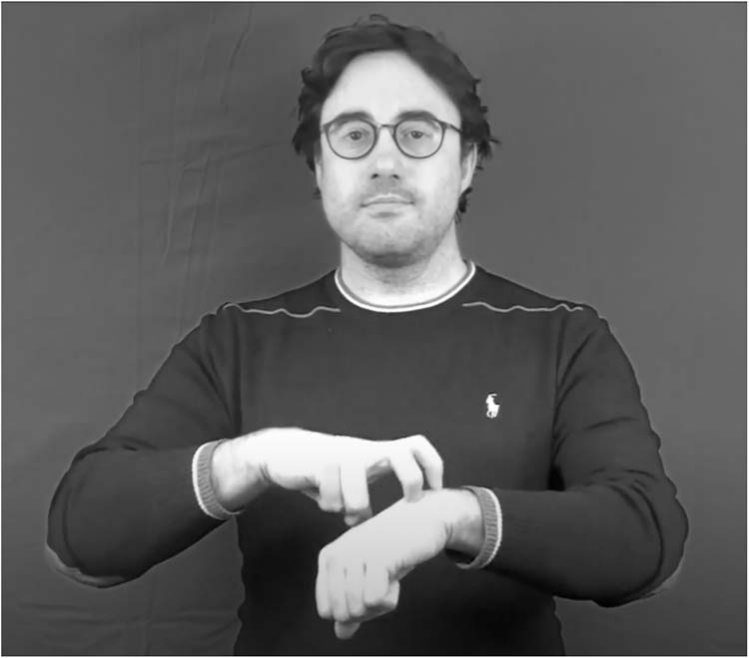
The sign doctor in LIS.

The *Lexical comprehension task with semantic distractors* (cf. Zorzi, Aristodemo, Cecchetto, & Donati, 2019; Zorzi, Giustolisi, Friedmann, et al., 2019 for LSF and LIS), like the one with phonological distractors, is a sign-to-picture matching task. For the two sign languages studied, this test aimed at obtaining a 100% overlap in the target and distractor items. Each version of the test included 18 target signs. Each item was presented with eight pictures, one corresponding to the target, and seven to semantic distractors, that is, signs that are close semantic competitors of the target. Given that this test was developed having in mind a possible adaptation to deaf signers with language impairments, the high number of semantic distractors was meant to favor the detection of impairments: the higher the stress induced by the sign semantic neighborhood, the stronger the chance to detect a semantic impairment in comprehension (Friedmann & Coltheart, 2017). Among the seven distractors, one was chosen to be also visually related to the target. For this distractor, we identified a visual relation between the form of the target sign and the concept of the distractor. For example, in LIS, watchmaker was selected as a visually related distractor for the target doctor since both concepts belong to the semantic category “jobs,” and the articulation of doctor may remind that of a watchmaker, see Figure 4. The idea behind this choice is that, in case a lexical item cannot be accessed, participants might be more likely to select a picture corresponding to a meaning that could be inferred if the sign was taken to be transparent. The addition of this distractor was meant to control whether the participants were just guessing the meaning of the target, which might be a possibility in case of language impairment. Another characteristic of the distractors was that there was no significant phonological relation between the targets and the distractors, where a phonological relation is taken to be significant if the target and the distractor share more than two parameters with the target sign. As already mentioned, the task was built in order to have the same targets and distractors in the two sign languages, as well as the same pictures. The only variations across the two languages were restricted to three pictures that varied due to cultural differences (i.e., in the representation of a train, police, and bread), and to the visually related distractor.

**Figure 5:**
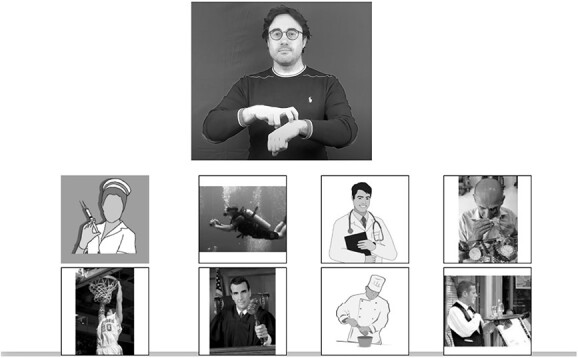
Example of one item of the Lexical comprehension with semantic distractors task in LIS (the original pictures in the test were in color).

Each participant watched the video of the target sign while the eight pictures were displayed and had to click on the matching picture (see Figure 5). The video could be watched only once. The test started with two training items. No filler was introduced.

#### Verbal agreement comprehension task

A compromised syntactic competence is one of the characteristics that can be displayed by individuals with language impairments. It is well known that linguistic structures involving syntactic movement, such as content questions (also called *wh*-questions), relative clauses, or passives, are those who are more susceptible to be impaired in any language deficit involving grammar (Friedmann & Shapiro, 2003; Grodzinsky, 1989, 2000; Grodzinsky et al., 1999, among others). Within the SIGN-HUB project, we designed four syntactic tasks to assess linguistic areas that can be compromised due to language impairments: comprehension of content questions, of relative clauses, of role shift, and of verb agreement. The general aim of these tests was to explore the importance of considering AoE as a factor to establish different baselines in language assessment, for either linguistic research or clinical purposes. The tests on content questions, relative clauses, and role shift (Aristodemo et al., 2022; Cecchetto et al., 2022; Hauser et al., 2021; Hauser et al., 2023*)* turned out to be fairly difficult, especially for non-native signers. On the contrary, the results of the test on verbal agreement were generally good, and this makes it a potential candidate for a clinical assessment test.

Agreement verbs are visually characterized by a movement between two loci in space associated with two or more arguments (e.g., the movement goes from the locus in space assigned to one argument, usually the subject, to the position in space of another argument). Hence, the direction of the movement depends on the locus of the arguments (Padden, 1983; Pfau et al., 2018, among others). The nature of verb directionality in SLs has been a topic of wide debate. While some scholars insist on the fact that verb directionality is a syntactic phenomenon and consider it as an expression of agreement, as we also assume, others insist on its gestural nature (for more details about these two theoretical positions, see Pfau et al., 2018, and Schembri et al., 2018).

Behavioral studies have shown that comprehension of verb directionality is affected by the AoE in ASL and BSL (Emmorey et al., 1995; Cormier et al., 2012b, among others), making them important parameters to include in our test design.

The *Verbal Agreement Comprehension Task* (Aristodemo et al., 2019 for LSF, Sala et al., 2019 for LIS) was a truth-value judgment task. Participants watched a brief non-linguistic clip showing three characters (A, B, and C) interacting, followed by a sentence containing an agreement verb. Participants had to judge whether the sentence matched the situation described in the clip (match condition) or not (mismatch condition). In the mismatch condition, the sentence could describe the correct situation but attributing inverted thematic roles to the characters or could display wrong argument selection (these two possibilities were balanced within the mismatch condition, and this dimension was not considered in the analysis). Control sentences, which were clearly wrong since they referred to a different situation, were added to assess participants’ understanding of the task.

Sentences were always signed by character A, who was therefore the grammatical first person, to character B, the grammatical second person. Character C was always associated with the third person. The three characters were the same across all stimuli.

Before the beginning of the task, participants were shown a video with character A signing the instructions. During the instructions, character B and character C were introduced (and they received a sign-name, as also character A did).

**Figure 6:**
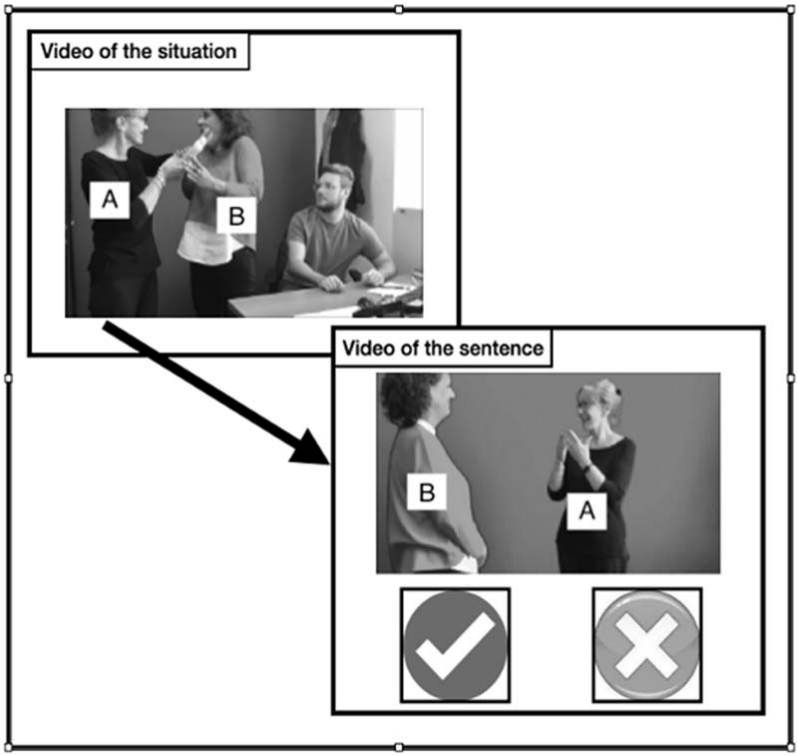
Example of the procedure in the Verbal Agreement Comprehension Task in LIS (the original videos and pictures in the test were in color).

We provide an example showing two screenshots of an LIS item. In the LIS example presented in Figure 6, character A (Anna, the woman with the back shirt) enters the room and gives a book to character B (Rita, the woman with the red shirt). A second video follows in which character A signs a sentence to character B that could match or not the situation previously seen. The matched sentence (cf. (1a)) was “I gave you a book”, whereas the mismatched sentence (cf. (1b-1c)) was either “You gave me a book” or “I gave a book to Pietro” (Pietro is character C). In the first case, there is an inversion in the directionality of the verb with respect to the target sentence, in the second case, a different argument is introduced. In this example, the control sentence (cf. (1d)) was “I gave you a flower.” The participant needed to click on the green button if the target sentence matched the non-linguistic situation and had to click on the red cross if there was no match.

(1) a. ix-1 book 1-give-2           (LIS)

“I gave you a book.”

b. ix-2 book 2-give-1.

“You gave me a book.”

c. ix-1 pietro_a_  book 1-give-3_a_.

“I gave a book to Pietro.”

d. ix-1 flower 1-give-2.

“I gave you a flower.”

In LIS, the task included 18 situations and 4 sentences for each situation: 1 correct, 2 mismatch (1 argument mismatch and 1 directionality mismatch) and 1 control, for a total of 72 sentences. Eighteen verbs were used. The 72 sentences were divided into four lists of 18 items each, with one occurrence of each situation in each list. The task was administered in two blocks, composed of two lists each. Each block started with two extra items as training (one correct and one with mismatched agreement). Within a block, the two lists were shown one right after the other, and within each list, items were randomized. For most of the participants, the two blocks were administered during the same experimental session, separated by at least three other linguistic tasks. Some participants received the two blocks in two different days.

In LSF, the task included 24 situations and 2 sentences for each situation (1 match and 1 mismatch, which could be either directionality or argument mismatch) and 20 control sentences, for a total of 68 sentences. Twenty-one verbs were used (three of them were repeated twice). Four sentences were used as training items. The remaining 64 sentences were divided into two lists so that each target situation was present only once in each list. Each list was administered in a single block; each block was presented on a different day during a wider experimental session including other tasks.

### Results and Analyses

All analyses were realized separately for each task and for each sign language using the R software (R Core Team, 2020). In all analyses, we considered accuracy as the dependent variable, binary-coded (correct answers were coded as “1” and incorrect answers as “0”). Among the various independent variables, we considered AoE group and also chronological age, as it was not homogenous in our three groups of signers (native, early, and late).

Models were implemented through the glmer function (package lme4, Bates et al., 2015), with the only exception of the lexical comprehension with semantic distractors task in the LIS analysis (see below) for which we ran a simpler glm analysis.

#### Lexical Comprehension With Phonological Distractors Results

The analyses were performed considering all items in LIS, whereas for LSF, four items were removed because accuracy was below 50%.

**Figure 7:**
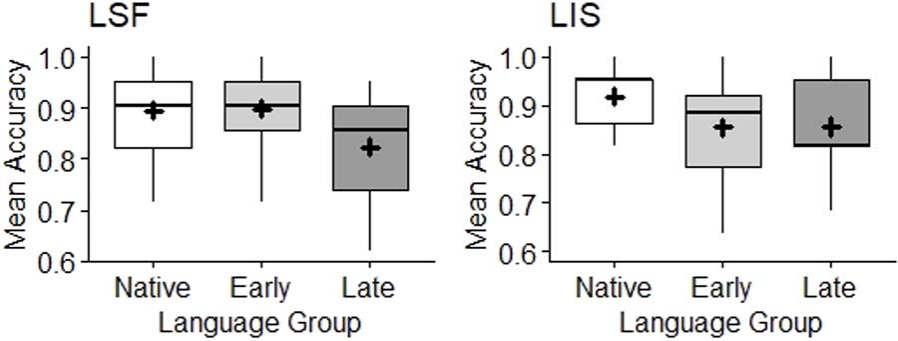
Mean accuracy across age of exposure (AoE) groups (native on the left, early in the middle, and late on the right). The mean for each group is marked through the black cross; the median is the horizontal black line. French Sign Languge (LSF) results (*n* = 43) are on the left; LIS results (*n* = 42) are on the right.

In the generalized linear mixed model analysis, we included AoE group (native, early and late) and age (mean centered continuous variable) as independent variables. Items and participants were inserted as random variables. Results are presented in Figure 7, and the summary of the statistical model is presented here after.

In LSF, age of participants was not a significant predictor of accuracy (β = −.009, SE = .01, z = −.65, *p = .51*). The performance of late signers was significantly worse than that of early signers (β = −.74, SE = .37, z = −1.98, *p = .047*), and it was worse than that of native signers, even if in this case, the difference did not reach significance (β = −.72, SE = .37, z) = −1.93, *p* = .053). On the contrary, the performance of native and early signers was comparable and, in fact, they did not differ significantly (β = .02, SE = .38, *z* = .06, *p = .95*).

In LIS, age of participants was a significant predictor of accuracy (β = −.05, SE = .02, *z* = −3.15, *p = .002*). Accuracy did not significantly differ between native and early signers (β = −.53, SE = .33, *z* = −1.58, *p = .06*), native and late signers (β = −.37, SE = .36, *z* = −1.01, *p = .31*), nor between early and late signers (β = .16, SE = .31, *z* = .51, *p = .61*).

All in all, when we consider mean accuracy across AoE groups, we see that participants were very accurate in this task. As such, a low performance in this task could reliably be interpreted as being the result of an impaired lexical phonological comprehension.

#### Lexical Comprehension With Semantic Distractors Results

The analyses were performed considering all items in LIS (*N* = 18), whereas two LSF items were removed because of technical problems resulting in 16 total items.

**Figure 8:**
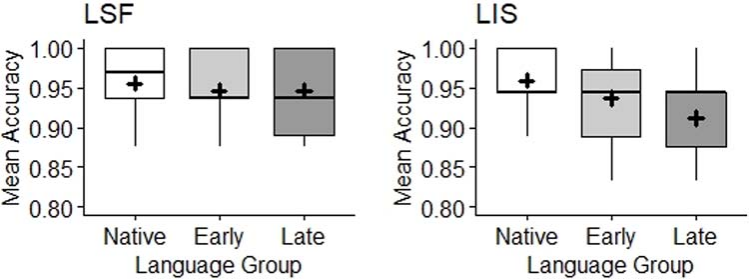
Mean accuracy across AoE groups (native on the left, early in the middle, and late on the right). The mean for each group is marked through the black cross; the median is the horizontal black line. LSF results (*n* = 43) are on the left; LIS results (*n* = 42) are on the right.

In the analyses, age and AoE group were inserted as independent variables. Items and participants were the random variables of our generalized mixed model. However, due to the very high accuracy scores across SLs and groups, and the relatively small size of the datasets (688 observations for LSF and 756 for LIS), such a complex model could not be computed, resulting in a singular fit. Following Barr et al.’s (2013) recommendation, we simplified the model by removing participants random variables in the LSF analysis and participant and items random variables in the LIS analysis, thus applying a simpler glm analysis. Results are presented in Figure 8.

In LSF, as Figure 8 shows, results were very similar across AoE groups with a mean accuracy of approximately 95% across AoE groups. The homogeneity of the signers’ performance irrespective of their age and AoE group is confirmed by the statistical analysis. The difference in accuracy between native and early signers was not significant (β = −.19, SE = .48, *z* = −.39, *p = .69*), as it is the case when comparing native to late signers’ performance (β = −.36, SE = .49, *z* = −.73, *p = .47*) and when comparing early to late learner signers (β = −.17, SE = .47, *z* = −.36, *p = .72*). The effect of age was not significant (β = .04, SE = .02, *z* = 1.76, *p = .08*).

In LIS, Figure 8 shows a progressive decrease in performance across AoE groups; however, all three populations displayed very high scores (around 95% again). This homogeneity is visible in the statistical analysis. The difference in accuracy between native and early signers was not significant (β = −.37, SE = .41, *z* = −.90, *p = .37*). The difference in accuracy between native signers and late signers was larger but again not significant (β = −.69, SE = .42, *z* = −1.64, *p = .101*). The comparison across early and late signers was not significant (β = −.32, SE = .35, *z* = −.90, *p = .37*). Finally, chronological age was not a significant predictor of accuracy (β = −.02, SE = .02, *z* = −.93, *p = .35*).

All in all, these results show a very good performance in deaf signing adults regardless of AoE or chronological age.

#### Verbal Agreement Comprehension Task: Results

Before analyzing the data, we removed those items in which native signers’ performance was below 50% accuracy, and we removed those participants whose score was below 75% accuracy in control items.

As for LSF, one item was removed, as well as four participants (one early signer and three late signers). In LIS, we removed one experimental item and one control item, and three participants (one native signer and two early signers).

**Figure 9:**
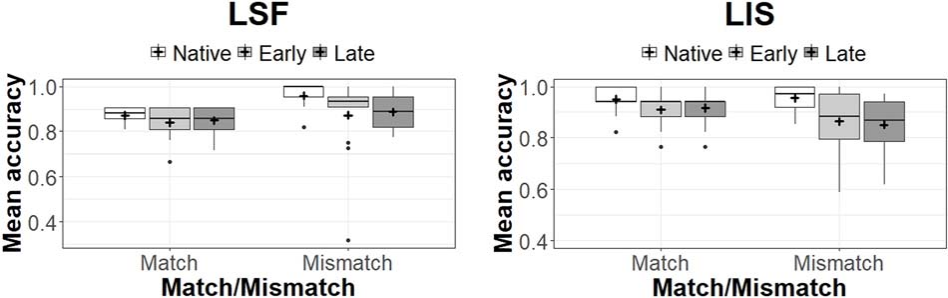
Mean accuracy across AoE groups (native on the left, early in the middle, and late on the right). The mean for each group is marked through the black cross; the median is the horizontal black line. LSF results (*n* = 40) are on the left; LIS results (*n* = 39) are on the right.

For the purpose of the present paper, the analysis will focus on the difference in accuracy between the three groups of participants in the two languages in the match/mismatch conditions (Figure 9).

In the glmer analysis, we entered group, condition (match/mismatch), chronological age (mean centered), and the group^*^condition interaction as fixed factors. To test if the interaction was significant, we performed likelihood ratio tests of the model with the interaction against the model without the interaction. We included in our models random intercepts for subjects and items.

Considering LSF, the interaction significantly contributed to the model’s fit (χ^2^(2) = 6.93, *p = .03).* Considering mismatch stimuli, natives outperformed both early (*p* = .0003) and late signers (*p = .002*), whereas for match stimuli, the difference between native and non-native signers was not significant. The difference between early and late signers was never significant. Moreover, for native signers, accuracy in match stimuli was significantly higher than accuracy in mismatch stimuli (*p < .001*). On the contrary, in the other groups, the difference between the two conditions did not reach significance.

As for LIS, the interaction term could be dropped without decreasing the model’s goodness of fit (χ^2^(2) = 3.33, *p = .19).* For all groups, accuracy in the match condition was higher than accuracy in the mismatch condition (*p = .01*), and across conditions, native signers outperformed both early and late signers (*p = .007* and .01, respectively). The difference in accuracy between early and late signers was not significant (*p = .99*).

#### Test–retest reliability

As mentioned in the section Sign Language Assessment Tools:  A Focus on Clinical Tests, while presenting the criteria that make a good assessment tool, an effective way to confirm the reliability of an assessment test is to measure over time through a test–retest (Kline, 2000) whether the scores obtained by the same subjects in two different occasions are correlated. For the two lexical tasks, a retest was administered to 16 LIS and 18 LSF participants a year after the first testing campaign. These numbers are due to the fact that only a subset of the participants accepted to participate in the retest campaign. Notice also that while the test was realized in a controlled environment (a lab), the retest was realized online due to the COVID-19 situation. Unfortunately, we were not able to collect retest measures for the agreement task. We recognize these as limitations in the results we are reporting. For the two sign languages, the participants that participated in the retest campaign were also involved in the first stage of the study. The same codes were used across both sessions to identify a given participant, hence ensuring maximal comparability in time.

According to Bland and Altman (1999), performing a simple *Pearson correlation test* is not enough to determine agreement of measure across tests. We thus performed the *Bland–Altman plot* on each sign language.

##### Results Test–Retest: Lexical Comprehension With Phonological Distractors

**Figure 10:**
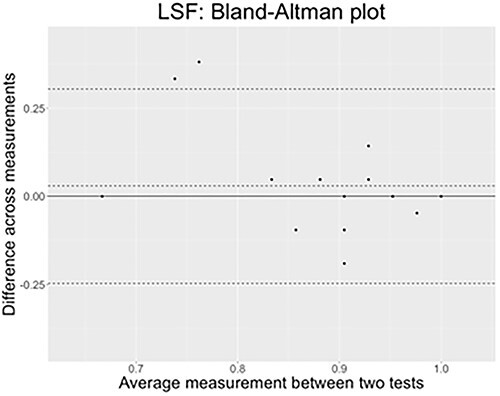
The *x*-axis of the plot displays the average measurement of the two tests, and the *y*-axis displays the difference in measurements between them (*n* = 18 comparisons). The continuous black line represents the average difference in measurements toward which a perfect agreement is reached (*y* = 0), while the three dashed lines represent the confidence interval limits for the average difference (external lines = limits of agreement) and the middle-dotted line represents the average difference that is effectively found in LSF results (*y* = .029).

We performed an analysis using the *blandr* package (Datta, 2017) on R software. Figure 10 presents the LSF results. As we can see, most data points lie within the confidence interval delimited by the two external dotted lines. The average bias is estimated to lie around .03, a very low score.

**Figure 11:**
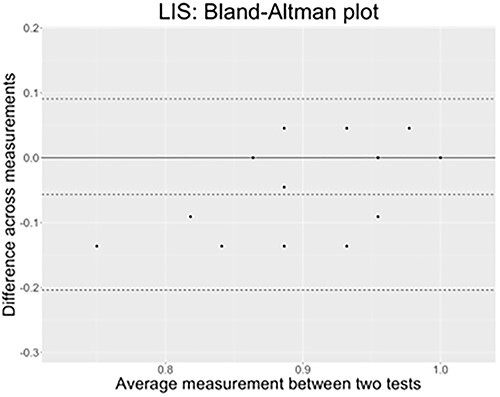
The *x*-axis of the plot displays the average measurement of the two tests, and the *y*-axis displays the difference in measurements between them (*n* = 16 comparisons). The continuous black line represents the average difference in measurements toward which a perfect agreement is reached (*y* = 0), while the three dashed lines represent the confidence interval limits for the average difference (external lines = limits of agreement) and the middle-dotted line represents the average difference that is effectively found in LIS results (*y* = −.057).

Figure 11 presents the LIS results. All datapoints are within the confidence interval, hence showing a high agreement between the two measures collected through the test–retest. Here again the bias estimated is very low (−.06).

With these results, we can confirm the reliability in time of the lexical comprehension task with phonological distractors.

##### Results Test–Retest: Lexical Comprehension With Semantic Distractors

**Figure 12:**
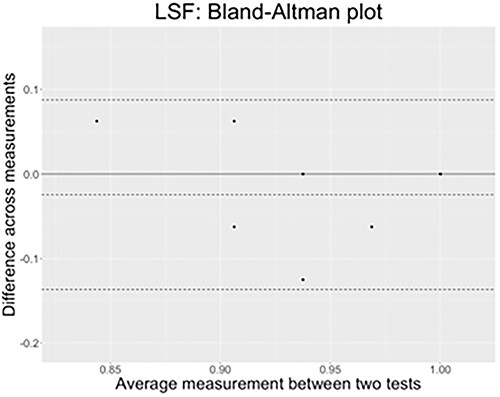
The *x*-axis of the plot displays the average measurement of the two tests, and the *y*-axis displays the difference in measurements between them (*n* = 18 comparisons). The continuous black line represents the average difference in measurements toward which a perfect agreement is reached (*y* = 0), while the three dashed lines represent the confidence interval limits for the average difference (external lines = limits of agreement) and the middle-dotted line represents the average difference that is effectively found in LSF results (*y* = −.024).

In Figure 12, concerning LSF, we can see that all datapoints lie within the confidence interval in LSF, and that the bias is only of around −.02 points. The test–retest performed on the lexical comprehension task with semantic distractors are agreeing across time.^5^

**Figure 13:**
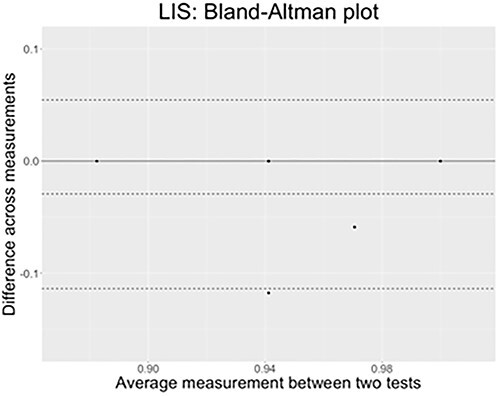
The *x*-axis of the plot displays the average measurement of the two tests, and the *y*-axis displays the difference in measurements between them (*n* = 16 comparisons). The continuous black line represents the average difference in measurements toward which a perfect agreement is reached (*y* = 0), while the three dashed lines represent the confidence interval limits for the average difference (external lines = limits of agreement); the middle-dotted line represents the average difference that is effectively found in LIS results (*y* = −.029).

In Figure 13, the LIS test–retest results show again a great agreement pattern with all points but one lying within the confidence interval delimited by the external dotted lines. The bias is also very low since it is of approximately −.3, hence confirming that measures taken the first time and 1 year later matches in this sign language as well.

In light of these data, we can say that the SIGN-HUB lexical tests are reliable tools to assess lexical comprehension with phonological and semantic distractors within each sign language we tested (LSF and LIS). They elicit very high performances in a typically developing population, irrespective of their age and AoE group, and the results obtained are stable in time as shown by the minimal biases values produced by the comparison of measures performed 1 year apart.

#### Further psychometric analyses

Given the applied purpose of the present work, that is, to identify materials that could be used in clinical settings, we performed a series of further analyses to evaluate the goodness of our items. Obviously, however, it will be only by using those tests with clinical populations that the validity of our materials in detecting language impairments will be eventually assessed.

As a first measure to evaluate the items’ quality, for each test, we computed the point-biserial correlation between item response and participants’ total score. Estimates of point-biserial correlation go from −1 to 1. Negative values indicate that participants who generally performed well scored badly in that item; therefore, items with negative estimates should be discarded. On the contrary, positive values indicate that participants who generally performed well also scored well in that item. Items with a negative coefficient should be better removed in a further version of the test, as indicated in the section Guidelines for Adaptation of the Three Tests.

Considering the lexical comprehension test with phonological distractors, there were items with a score of 100%, for which it was not possible to compute the point-biserial coefficient (LSF N = 3, LIS N = 7). Considering the remaining items, two had a negative coefficient in the LSF test (item *13-SALAD* and item *14-UGLY*) and one in the LIS test (item *12-SUN*).

As for the lexical comprehension test with semantic distractors, 10 LSF items and 9 LIS items obtained a 100% score. Only one LIS item had a negative point-biserial coefficient (item *01-DOCTOR*). Focusing on the verbal agreement comprehension task, 8 LSF and 10 LIS items obtained a 100% score. In both languages, four items had a negative coefficient (LSF: three items in the match condition, *ASK*, *STEAL1*, and *CHOOSE2*, and one item in the mismatch condition, *LOOK_AT*; LIS: three items in the match condition, *CALL_BY_TOUCHING*, *PUSH, NOTIFY*, and one item in the mismatch condition, *CRITICIZE*).

Considering a subset without these potentially problematic items, items were also evaluated using item response theory (IRT) and a two-parameter model. Even if our sample sizes are too small to ensure an accurate estimation of item location and discrimination parameters (De Ayala, 2009), we used the obtained estimations as rough indication of items’ difficulty and discriminability. This analysis was performed using the *ltm* R package (Rizopoulos, 2006).

As for the lexical comprehension test with phonological distractors, in LSF, two items (*20-LEMON* and *22-BUS*) have a negative discrimination. This is not to be expected in a test since it indicates that the probability of selecting the correct answer decreased as the participant’s ability increases. As for the other 17 items, they all have negative difficulty, thus indicating that all items are easy. As for LIS, three items (*02-EGG, 10-PEPPER,* and *19-WAKE_UP*) have negative discrimination, whereas the other 18 items with positive discrimination are all easy, with negative difficulty.

Considering the lexical comprehension test with semantic distractors, in LSF, three items (8*-STRAWBERRY, 12-WOLF,* and *14-THEATRE*) have negative discrimination and the remaining 13 items have all negative difficulty. In LIS, two items (*16-THEATRE* and *18-TOAST*) have negative discrimination and the remaining 15 items have negative difficulty.

Moving to the verbal agreement comprehension task, in LSF, three items had a negative discrimination coefficient (two in the match condition, *EXPLAIN* and *INFORM*, and one in the mismatch condition, *PROVOKE*). Of the remaining 36 items, only one had positive difficulty (*CHOOSE1*). As for LIS, no item had negative discrimination, and only one item (*INVITE*, mismatch condition) had positive difficulty.

All in all, the number of items that are potentially problematic turned out to be fairly small, especially considering that we are dealing with two understudied languages; therefore, the selection of the items involved new research on the lexicon, phonology, and syntax of the two languages. As we explain in the next section, the existing tests can be easily adapted into clinical ones.

### Guidelines for Adaptation of the Three Tests

The results of the three tests presented show a high score from a healthy population of deaf signers and therefore allow to conclude that these tests are easy for this population and might therefore be suitable to test deaf signers with language impairments. Moreover, the test–retest for the lexical tests confirms the validity of these two tests.

To assess items’ quality, further psychometric tests were run, such as point biserial correlations and item response theory analyses. The results of these tests, described in the previous section, provide different measures to detect potentially problematic items.

We used those results to provide guidelines on how to change the selection of items for the three tests presented in this work to turn them into tools to be used with a clinical population in the future. A first version of general guidelines on how to improve these three tests to become clinical tests were given in Friedmann et al. (2020), but no analysis of the items was conducted at the time. Based on the psychometric tests, future versions of these three tests should not include the items with a negative point biserial coefficient nor those with negative discrimination. The materials of the existing tests will be available at the OSF repository for LIS and LSF, and, together with the guidelines provided in this section, it will be possible to administer the two lexical assessment test and the syntactic one on verbal agreement to a clinical language–impaired population.

#### Lexical tests

Starting with the lexical comprehension test with phonological distractors, in LSF, the four items *13-SALAD*, *14-UGLY, 20-LEMON,* and *22-BUS* should be removed; in the LIS test, the four items *02-EGG*, *19-WAKE UP*, *10-PEPPER,* and *12-SUN* should not be included in the final clinical test.

In the lexical comprehension test with semantic distractors, as well, a number of items should be removed: for LSF, the items *8-STRAWBERRY*, *12-WOLF,* and *14-THEATRE*; for LIS, the three items *01-DOCTOR, 16-THEATRE,* and *18-TOAST*.

Given the complementary nature of these two tests in assessing lexical impairment, the two tests were built to tackle phonological and semantic impairment separately. A suggestion is to use the results of both tests in order to get a global evaluation of the potential lexical impairment of the patient.

#### Verbal agreement test

The size of the syntactic verbal agreement test will need to be generally reduced. As presented in the section Verbal Agreement  Comprehension Task, this test had four conditions for each item: matched (the correct sentence), mismatch with inversion of the directionality of the verb, hence of the arguments, mismatch realized with the use of a different argument of the verb, and a control condition where the direct object was changed. The general suggestion is to remove the control items and to make sure to have an equal number of items for the two mismatch conditions.

As for the items to be removed, based on the items with a negative point biserial coefficient or with negative discrimination, in the final LSF test, it will be necessary to remove the items *ASK, STEAL1, CHOOSE2, EXPLAIN,* and *INFORM* in the matched condition, and *LOOK AT* and *PROVOKE* in the mismatched condition; in the LIS tests, the items *CALL_BY_TOUCHING, PUSH,* and *NOTIFY* should be removed in the matched condition and *CRITICIZE* in the mismatched condition*.*

## Discussion and Conclusions

The SIGN-HUB lexical comprehension tasks and the test for assessing comprehension of verbal agreement have good potential to become valid clinical tests. Following the criteria listed by Haug (2005), the tests presented in this work are, in the first place, language specific and the choice of items was based on linguistic research, and they were selected in collaboration with deaf sign language experts; second, the general results of their first administration to healthy populations of signers in France and Italy showed an overall good performance in the three groups of healthy participants; third, the lexical tasks were test–retested and showed a strong correlation of the results over time.^6^ Importantly, the psychometric tests run to measure items’ quality provide the data necessary to determine how to adapt the tests in order to increase the goodness of the tests. The results allowed us to detect the items to be removed and to formulate some guidelines on how to adapt the tests into clinical tests. It is important to underline that the data reported here come from a healthy population of signers and that the validity of three assessment tools as clinical tools need to be tested with a sample of impaired signers. This aspect is a clear limitation of the work presented here. Another limitation is related to the verbal agreement test where no test–retest was run. However, we believe the materials and the guidelines we are making available are a solid starting point for the creation of clinical tools to test language impairments.

Focusing on the overall general performance of the healthy participants presented in this work, there are important aspects concerning AoE effects that need to be addressed. A different pattern was found between the syntactic test and the lexical ones. In the *Verbal agreement comprehension task*, accuracy was higher for native signers compared to both groups of non-native signers both in LIS and in LSF. This pattern aligns with the results found for other LIS and LSF tests assessing comprehension of other syntactic structures studied within the SIGN-HUB project.^7^ On the contrary, AoE had a different (and overall very weak) impact on the results of the lexical tasks. We observed significant effects of AoE group only in the *Lexical comprehension with phonological distractors task* in LSF, where the significant difference was only between late and native signers. This is in line with various studies showing no differences in accuracy related to the AoE in lexical comprehension tasks, suggesting that the AoE does not have a major role in determining vocabulary size (e.g., Dye & Shih, 2006; Carreiras et al., 2008; please notice that in these and other studies, despite no difference in accuracy, native signers performed lexical decisions faster than non-native signers). A difference, though, might be found in clinical settings because a native lexicon might be more resistant to damage (this hypothesis requires further investigation within a clinical population, which is outside the scope of the present paper).

The pattern that we observed with the syntactic verbal agreement task, on the other hand, is also in line with previous literature that shows a categorical difference between native and non-native signers and not between early and late signers (for an overview, see Zorzi et al., 2022). In the verbal agreement test, early and late signers performed lower than native signers both in LIS and LSF (in both the match and mismatch conditions in LIS and only in the mismatch condition in LSF), confirming that comprehension of verbal agreement can indeed be affected by the AoE in sign languages, as already found by Emmorey et al. (1995) and Cormier et al. (2012a), among others. These data on LIS and LSF suggest that different normative data based on the AoE are necessary when assessing syntactic competence in deaf signers. The use of specific baselines relates to the need to clearly identify reference groups for which the test is to be standardized, taking into consideration the heterogeneity of the deaf population (Haug & Mann, 2008). As it is well known, the deaf population is characterized by many different linguistic backgrounds, and native signers, defined as deaf individuals who were born into a deaf signing family, are only a small minority. It is thus crucial to have a good understanding of the linguistic profile of native and non-native signers, whose categorical difference has been shown to have a potential impact on the grammar itself. The case of Catalan Sign Language non-native signers, who do not appear to have object relative clauses in their grammar, is an extreme example (for a detailed discussion about this, see Hauser et al., 2021). Moreover, setting different baselines for different populations will make sure that the diagnosis of a patient is adequate to their potential level of language competence, eventually ensuring a more effective intervention plan. A categorial difference between native and non-native signers when assessing syntax has a concrete consequence for the verbal agreement test presented here. It implies that, once the test has been adapted for clinical use, it needs to be run with a large population of heathy signers to establish the thresholds that allow to properly assess native and non-native signers and even more impaired signers.

While we are aware of the limitations that come with the materials that we are making available, we would like to highlight that the SIGN-HUB tests presented in this paper constitute an important step toward the construction of clinical assessment tools for adult signers in languages that lack them entirely.

## Endnotes

^1^In some cases, parts of tests created with different purposes are implemented into one another, like in the case of some parts of the *Test Battery for ASL Morphology and Syntax* (Supalla et al., 1995) that were used in the *American Sign Language Assessment Instrument* (ASLAI) and in the *American Sign Language-Proficiency Assessment* (ASL-PA).

^2^Some of the assessment tests presented in this section target the same grammatical aspects studied in Atkinson et al. (2005), but they were not built to be used in clinical settings and they are meant to target only children. The *Phonological judgment task* that is used in the *ASL phonological awareness test (ASL-PA)*, or *The Developmental Assessment Checklist for Sign Language of the Netherlands* (NGT-OP) are phonological tests that involve the use of phonological distractors through the selection of minimal pairs. The NGT-OP test also assesses the lexicon from a semantic perspective, but it does it only in production using a structured questionnaire. As for the assessment of agreement verbs, the ASL-PA, the ASLI, and the *Assessment of Sign Language of the Netherlands* (Jansma et al., 1997) look into these linguistic features, but they do it mainly analyzing production and anyway targeting children.

^3^See Aristodemo et al. (2022), Hauser et al. (2023), Cecchetto et al. (2022), and Hauser et al. (2021) for the description of the tests on role-shift, wh- clauses in LSF, wh- clauses in LIS and relative clauses, respectively. These papers also present data analysis and discussion.

^4^To control for (a), we followed the indications of our deaf consultants. To verify the level of transparency of a sign and its representability with a picture, several validations were administered to hearing non-signers from the same country of the sign language assessed. Pictures were also validated. For more detailed information on the validations of the signs and pictures, please consult Friedmann et al. (2020).

^5^Note that the number of observations is still of 18 but many points overlap.

^6^As explained in the section Test–Retest Reliability, we did not run a test–retest for the verbal agreement test. While the lexical tasks will not be changed much in their clinical version, the agreement test needs to be importantly reduced, as explained in the section Guidelines for Adaptation of the Three Tests. The results from the IRT analysis on the items show anyway that the materials were well selected.

^7^See Hauser et al. (2021) for the impact of AoE in the comprehension of relative clauses, Aristodemo et al. (2022) in the comprehension of role shift, Cecchetto et al. (2022) for the comprehension of wh-questions in LIS, and Hauser et al. (2023) in LSF.

^8^For LSF, one native and five early and three late signers declared to use LSF “often” instead of “everyday.”

## Funding

SIGN-HUB project—European Union’s Horizon 2020 Research and Innovation Program (Grant Agreement N 693349).

## Conflicts of Interest

No conflicts of interest were reported.
